# Guidelines for Evaluating Clinical Research Training using Competency Assessments

**DOI:** 10.15694/mep.2019.000202.2

**Published:** 2020-09-22

**Authors:** Elias Samuels, Phillip Anton Ianni, Haejung Chung, Brenda Eakin, Camille Martina, Susan Lynn Murphy, Carolynn Jones

**Affiliations:** 1 Michigan Institute for Clinical and Health Research; 2 Michigan Institute for Clinical and Health Research; 3 Tufts Clinical and Translational Science Institute; 4 Tufts Clinical and Translational Science Institute; 5 Clinical Translational Science Institute; 6 Clinical Translational Science Institute; 7 Center for Clinical and Translational Science; 8 Center for Clinical and Translational Science

**Keywords:** clinical and translational research, workforce development, competency-based assessment, competency framework, program evaluation, program improvement, logic model

## Abstract

This article was migrated. The article was marked as recommended.

Effective training programs in clinical and translational research (CTR) are critical to the development of the research workforce. The evolution of global CTR competencies frameworks motivates many CTR institutions to align their training offerings with these professional standards. Guidelines for integrating competency-based frameworks and assessments into rigorous program evaluations are needed in order to promote the quality and impact of these training programs. These guidelines provide practical suggestions for how to ensure that subjective and objective assessments of CTR knowledge and skill can be effectively integrated in the evaluations used to improve these essential training programs. The approach presented here necessarily involves the systematic and deliberate incorporation of these particular types of assessments into comprehensive evaluation plans. While these guidelines are broadly applicable to the work of those charged with developing, administering and evaluating CTR training programs, they have been specifically designed for use by program directors.

## Introduction

Clinical and translational research in the United States is supported by numerous federal, industrial and academic organizations, and many other stakeholder groups ( Callard, Rose and Wykes, 2012; Martinez *et al*., 2012; Trochim, Rubio and Thomas, 2013; Joosten *et al*., 2015). The NIH National Center for Advancing Clinical and Translational Science (NCATS) is a distinctive among them as it funds a broad network of research support centers, Clinical and Translational Science Awards (CTSAs), embedded in over 50 research institutions located across the country ( NCATS, 2018). A key strategic goal of these CTSAs regards the development of the clinical and translational workforce through dedicated research training programs ( NCATS, 2017). Clinical Translational Research (CTR) training programs provide highly valued instruction on relevant research skills and demand rigorous evaluations that demonstrate their impact on the development of the research workforce ( Bonham *et al*., 2012; Calvin-Naylor *et al*., 2017).

CTSA programs offer a variety of training options, typically in the form of short-term programs, short courses or one-time workshops. These training programs are often tailored to the need of professional degree students on a research tracks, postdoctoral fellows, residents, or early career faculty. These programs often provide education and training in a variety of core competencies, including study design, communication, teamwork, and research ethics, to name only a few areas of study.

Rigorous evaluations of CTR programs periodically require measurement of demonstration and application of research skills and acquired competencies ( Misso *et al*., 2016). Medical education programs are often subject to quality control, quality management, and quality assurance by regulators. However, no analogous formal mechanism exists for evaluating CTR programs. Instead, the responsibility for evaluating CTR education and training programs often resides with small groups of investigators, research managers and administrators with little experience measuring research competencies per se. This work provides concrete steps they can take to integrate competency assessments into evaluation plans implemented by CTR training programs ( Centers for Disease Control and Prevention, 1999; Trochim, Rubio and Thomas, 2013).

In this paper, we provide twelve guidelines for evaluating research education and training programs to better understand learner attainment of the skills and knowledge in clinical translational sciences. The guidelines discussed in this paper have been adapted to the role of the CTR training program directors. Therefore, to ensure the relevance of these guidelines to this role, the authors carefully considered the typical demographics, job duties, motivations, knowledge, skills and experiences of an administrator charged with guiding the evaluation and quality improvement of these education and training programs.

## Guidelines for using competency assessments in program evaluation

### Review team roles and expertise related to trainee’s professional development

The responsibility for evaluating CTR training programs is often carried out by personnel in a number of positions and roles. The collaborative review of these roles can be facilitated by creating *personas*, which are defined as archetypes with distinctive needs, goals, technical skills and professional characteristics ( Adlin and Pruitt, 2010). Creating a persona that defines who will be conducting evaluations can help program teams and stakeholders discuss and negotiate changes to the ways this work is distributed and coordinated. Table 1 provides examples of personas of clinical research professionals who are likely to share responsibilities for administering a CTR training program. This process can be carried out by CTR program leads and administrators to help focus collaborative efforts on measuring the research knowledge and skills of researchers.

**Table 1.** Professional roles involved with evaluating Clinical and Translational Research (CTR) training programs

Persona			
	CTR Investigator	CTR Training Program Director	CTR Training Program Administrator
**Associated professional responsibilities**	Junior investigators or senior research fellows	Research department supervisor or supervisor of training programs for research team members and junior investigators	Research and regulatory support or program manager
**Professional motivation**	Wants to provide rigorous training for research teams who are required to complete research training.	Wants to use assessments of clinical research skill to revamp educational programs.	Wants to provide consistent training and professional development experiences for research teams
**Understanding of best practices in evaluating learning**	Expertise in program evaluation, use of logic models and postsecondary teaching	Understanding of learning outcome assessment, CTR competency frameworks and postsecondary teaching	Understanding of survey administration, data management and use of observation checklists
**Responsibility for competency assessment administration, analysis and reporting.**	Identifying validated competency assessments and interpreting the results with stakeholders	Developing assessment forms and communicating with CTR trainees and developing results reports for stakeholders	Communicating instructions to CTR trainees and instructors, monitoring administration of assessment forms and management of resultant data.

### Integrate competency frameworks into evaluation planning

Ideally, evaluators should be involved in the process of developing training programs to identify learning outcomes. However, early involvement may not always be possible due to contextual constraints. Ideally evaluators should be involved in mapping any existing CTR training curriculum to *competency-based education* (CBE) frameworks ( Dilmore, Moore and Bjork, 2013). It may be necessary to partner with subject matter experts who understand CBE during this mapping process. There are multiple evidence-based competency frameworks applicable to CTR education and training ( NCATS, 2011; Calvin-Naylor *et al*., 2017; Sonstein *et al*., 2018). Table 2 shows training opportunities that have been mapped to one domain of an established CTR competency framework ( Joint Task Force, 2018).

**Table 2.** Sample Training Offerings for Scientific Concepts and Research Design

Developing Research Questions	Choosing an Appropriate Study Design	Selecting Valid Instruments	Determining an Adequate Number of Study Participants
Developing and Writing Research Questions, Aims & Hypotheses Formulating Research Questions, Hypotheses and Objectives The use of hypothesis testing in the social sciences	Experimental & Observational Study Designs Introduction to Clinical and Translational Research: Study Population and Study Design The Qualitative Research Process: Study Designs for Health Services Research	Finding Tests & Measurement Instruments: Library Research Guide Measuring assessment validity and reliability Community engaged approaches to measuring study team dynamics	Hypothesis Testing: Significance level, power, and basic sample size calculation Introduction to Power in Significance Tests Best practices in participant recruitment

The outputs of this mapping process should be shared with programmatic stakeholders to facilitate the collection of their feedback about the breadth and depth of the existing or potential CTR training opportunities. Collecting stakeholder feedback about the content of CTR training programs is an essential first step in many guides to evaluating health research training programs, including the U.S. Center for Disease Control and Prevention’s (CDC) guide for the evaluation of public health programs ( Centers for Disease Control and Prevention, 1999).

### Engage stakeholders in identifying critical knowledge and skill outcomes

As soon work on an evaluation plan has begun, evaluators should engage program stakeholders to help identify the most important knowledge and skills taught to CTR trainees. In collaboration with various stakeholder groups, evaluators can partner with instructional designers and other stakeholder groups to develop relevant and measurable lists of competencies. When identifying which specific stakeholder groups to involve in this phase of the evaluation planning process it is important to ensure that those with divergent recommendations of which CTR skills are in greatest need of development and assessment are included ( Callard, Rose and Wykes, 2012; Martinez *et al*., 2012; Trochim, Rubio and Thomas, 2013; Joosten *et al*., 2015).

Diverse stakeholder feedback can be systematically collected and synthesized using standard survey methods, interviews, focus groups and Delphi panels ( Brandon, 1998; Geist, 2010). Evaluators should collect stakeholder opinions about short- and long-term outcomes, including those regarding participant learning and behaviors ( Kirkpatrick and Kirkpatrick, 2006). The collection of data on all types of programmatic outcomes, but particularly including the knowledge and skills accrued through the program, is necessary for the development of rigorous program evaluations ( Centers for Disease Control and Prevention, 1999; Trochim, Rubio and Thomas, 2013). Evaluators should take care to consider all program characteristics relevant to key outcomes, most particularly those affecting the learning environment in which learners and their instructors are expected to work.

### Develop models depicting the links between program operations and outcomes

Logic models should be created in order to enrich and advance conversations with stakeholders and other administrators about the operation and impact of a CTR training program. Logic models are figures that typically depict the relationship between key programmatic A) inputs, B) activities, C) outputs and D) outcomes, often using itemized lists arranged into columns under each of these headers ( McLaughlin and Jordan GB, 1999). Some also include references to relevant important contextual or environmental factors affecting key programmatic goals. The choice of which elements to represent in the model should be informed by the need to visualize links between programmatic operations and skills development that would be of greatest interest to key stakeholders. Many funders ask that logic models be included in program proposals, and the production of these figures are standard practice in the evaluation of training programs in the health sciences ( Centers for Disease Control and Prevention, 1999; 2018).

**Figure 1.**
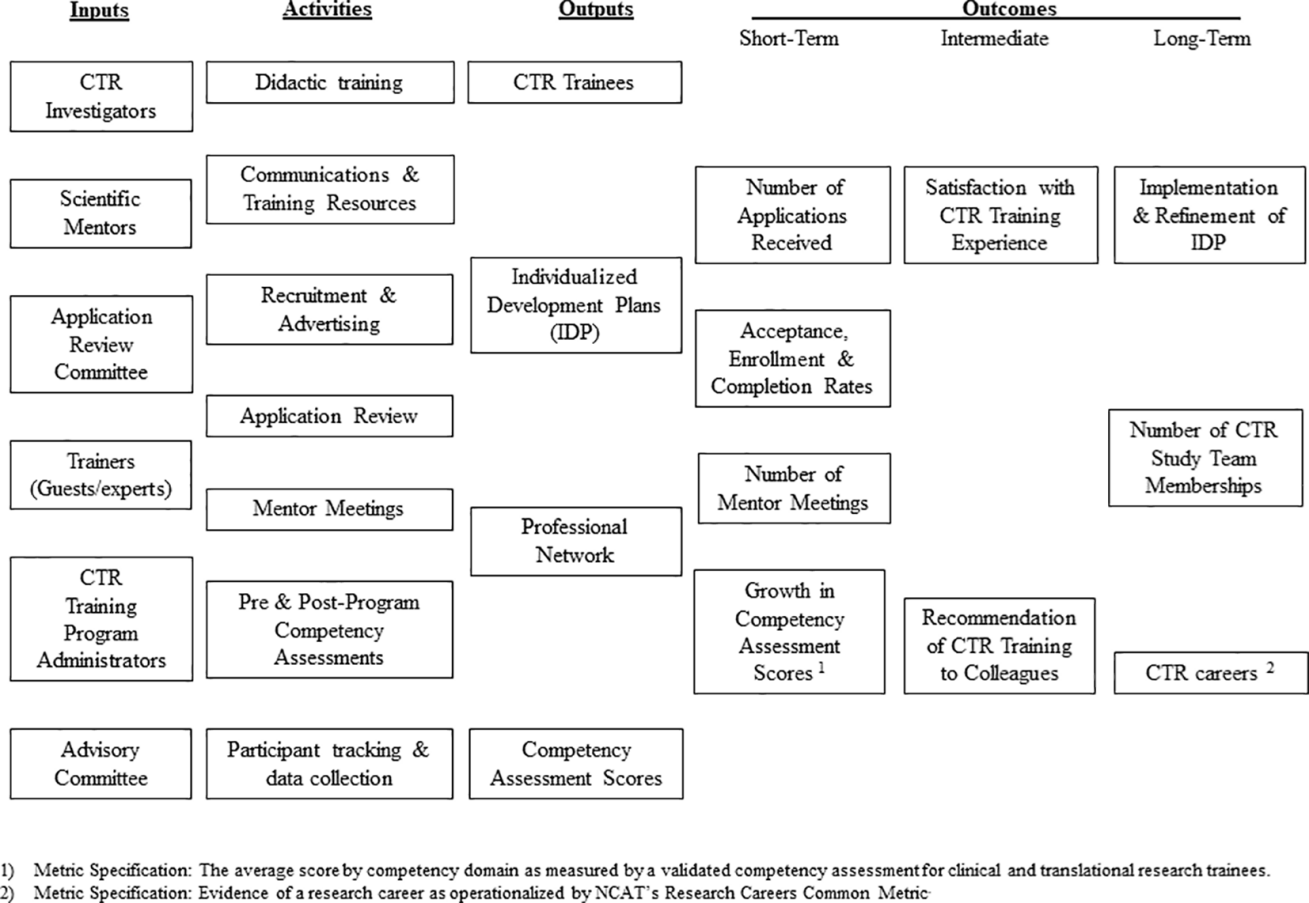
Sample Logic Model for an Evaluation of a CTR Training Program

Whenever possible, logic models for CTR training programs should include the identification of short-, intermediate-, and long-term goals. The acquisition of critical research knowledge and skills are often represented as outputs or short-term outcomes of these training programs in logic models. In contrast, distant impacts, such as the production of research grants, peer-reviewed publications and career advancement are often represented as intermediate- or long-term outcomes. To enhance the efficiency of the model-making process, utilize competency domains, each of which cover sets of related competencies, as outcomes rather than numerous specific competencies in these figures. Exemplars of logic models that include lists of CTR competency domains have been published and can be used inform development of logic models for similar programs ( Rubio *et al*., 2010). Figure 1 shows a logic model that can be used a basic template for enabling the planning, implementation and evaluation of a CTR training program.

### Distinguish programmatic outcomes used for formative and summative evaluation

The choice of when to measure outcomes should be informed by the intent to use the results for formative or summative evaluations ( Newman *et al*., 1995; Yudkowsky, Park and Downing, 2019). Formative evaluation is typically conducted during the development or improvement of a program or course, whereas summative evaluation involves making judgments about the efficacy of a program or course at its conclusion. The results of formative evaluations are often used to improve programs and projects during their implementation. Outcomes chosen for the purpose of formative evaluation are often represented as short- or intermediate-term outcomes in logic models. The results of summative evaluations are used to produce valid and objective measures of programmatic impact at the end of the implementation process. Footnotes can be added to logic models to differentiate the use of certain metrics for these two distinct purposes, as shown in the template above ( Figure 1).

Measures of knowledge and skill can be used for both the formative and summative evaluation of CTR training programs. The results of relevant pre-program assessment tools, and of assessments conducted during the course of a program, enable formative evaluation when they are used to improve the experience of the currently participating trainees. For example, the results of subjective or objective skill assessment tools can be shared with respondents to inform development of individualized training plans or used to inform modifications to training curricula to address perceived or objectively-measured gaps in knowledge and skill. The results of post-program skill assessment tools enable summative evaluation when compared to relevant benchmarks, including the results of pre-program skill assessment tools or measures of skill acquisition produced by similar training programs ( Newman *et al*., 1995; Centers for Disease Control and Prevention, 1999).

### Select validated assessment tools to measure critical knowledge or skills

The validation of knowledge or skill assessment tools requires that evidence be marshalled to advance the claim that an assessment tool actually measures what it was designed to measure ( Kane, 1992). Peer-reviewed publications that demonstrate the validity of a competency-based assessment will include the results of tests suggesting that the assessment tool provides reliable and accurate measures of knowledge or understanding among the level of learners targeted by the training program. The use of validated competency-based assessment tools for program evaluation lends credibility to the work and typically requires fewer resources than does the development of locally-developed assessments.

Several validated tools of CTR skills for investigators and research professionals have been published in recent years ( Bakken, Sheridan and Carnes, 2003; Streetman *et al*., 2006; Bates *et al*., 2007; Ellis *et al*., 2007; Mullikin, Bakken and Betz, 2007; Lowe *et al*., 2008; Cruser *et al*., 2009; Cruser *et al*., 2010; Lipira *et al*., 2010; Murphy *et al*., 2010; Poloyac SM *et al*., 2011; Robinson *et al*., 2013; Ameredes *et al*., 2015; Awaisu *et al*., 2015; Robinson *et al*., 2015; Sonstein *et al*., 2016; Jeffe *et al*., 2017; Patel *et al*., 2018; Hornung *et al*., 2019). When choosing between validated assessment tools, it is critical to select ones which are most closely aligned with the competency framework chosen for a given CTR program and which have been validated using learners with similar credentials and research experience to those participating in that program. Be sure to obtain all the necessary permissions from the creators of any validated assessment tools before using the instruments for evaluation purposes.

### Subjective vs. Objective assessment tools

The design and purpose of a CTR training program may require the use of subjective and objective assessment tools. Subjective assessment tools, through which participants rate their own knowledge or skills, can provide valid measures of self-confidence in one’s abilities, but have not been shown to correlate with the results of objective measures ( Hodges, Regehr and Martin, 2001; Davis *et al*., 2006). Subjective and objective assessments of CTR knowledge and skill can be used simultaneously, but only the results of the latter type should be used to make justify claims about the actual knowledge and skills currently possessed by CTR participants.

Clinical and translational research training programs that confer any level of certification which are formally recognized by professional institutions or organizations may require that objective assessment tools be used to verify the actual research capabilities of the graduates. In these cases, the specific assessment tools that should be used by CTR training programs may have already been identified by these associated professional groups. When multiple or conflicting assessment tools are required by these groups conversations with programmatic stakeholders will be needed before any final determination about the use of any competency-based assessment tools can be made.

### Estimate the time and effort required for implementing an evaluation plan

Evaluation plans take many different forms, but all plans detail how evaluation data will be collected, analyzed, reported and used ( Trochim, Rubio and Thomas, 2013). The costs of implementing rigorous evaluation plans can be substantial, so it is essential that they are accurately estimated and budgeted for. Some evaluation activities, such as the administration of publicly-available skill assessment tools using free online platforms, have comparatively low costs. The costs of other evaluations, such as those involving focus groups, can be considerably higher.

The effort required for each step of the evaluation plan can be estimated in a basic table ( Table 3). When reviewing an evaluation plan, carefully consider the risks and benefits of proposed assessment tools and choose those that are feasible to administer given the available financial and human resources. Collaborate with stakeholders to ensure that key evaluation activities are aligned with other project timelines and plans that guide the allocation of financial, human, and institutional resources needed to implement a CTR training program ( Centers for Disease Control and Prevention, 1999).

**Table 3.** Example evaluation activities and time required for an evaluation of CTR training

Evaluation Activities	Hours
*Evaluation Planning*	
Develop competency crosswalk for program components	4
Draft logic model with short, intermediate & long-term outcomes	4
Draft and submit IRB application	8
*Data Collection*	
Institutional records of participant affiliations	2
Competency assessment administration	4
Focus group administration	8
Focus group transcription	16
*Data Analysis*	
Cleaning and management of all quantitative data	4
Quantitative analysis of competency assessment data	8
Qualitative coding of focus group data	16
Qualitative coding of participant research projects	2
*Reporting*	
Draft Stakeholder Reports	40
**Total:**	116 hrs. (~3 weeks)

### Train evaluation team members to collect assessment data in reliable ways

Once an evaluation plan has been developed, and a formal evaluation team has been assembled, it is important that team members understand the steps required for reliable data collection using competency-based assessments. For example, use of a CTR assessment of regulatory compliance may require that all persons administering the assessment tool be consistent in their use of the instrument as well as their subsequent scoring of individual’s performance. Even objective scoring systems include risks related to subjective interpretations ( Van der Vleuten *et al*., 2010). Research has shown that individuals in apparent positions of power may influence or dissuade respondents from giving honest responses on tests of their knowledge or skills ( Taut and Brauns, 2003; Van der Vleuten *et al*., 2010). Therefore, it is essential that the appropriate team members receive and demonstrate their understanding of validity, reliability, evaluation ethics, conflicts of interest, possible hegemonic practices or biasing and reporting procedures.

### Use technology platforms that best facilitate data collection, analysis and reporting

Because no single technology platform specifically designed for CTR evaluation currently exists, evaluators must make use of existing platforms that are not tailored to CTR. To maintain consistency, accuracy and accessibility of the assessment results, CTR evaluators should use one platform to administer, analyze and report survey results whenever possible. For example, the same platforms used by clinical and translational researchers to collect research study data, such as REDCap ^TM^ ( Harris *et al*., 2009), Qualtrics®, and SurveyMonkey®, can also be used to conduct evaluations of CTR skills. If necessary, the resultant data can also be extracted from these platforms so that further analyses can be performed.

Many statistical analysis programs familiar to clinical and translational researchers, such as STATA, SAS and R, can also be used for rigorous validity tests. These software programs have the ability to conduct exploratory and confirmatory factor analysis ( Levine, 2005; Osborne and Costello, 2005), which are commonly used to identify and to validate the accuracy of the competency domains that structure many competency-based assessments. While there are many valuable validity tests ( Kane, 1992), these are the ones most commonly used to validate skill assessments. Software programs for qualitative analysis, such as Dedoose® or NVivo®, can be used to conduct qualitative evaluations of CTR programs ( Comeau *et al*., 2017).

### Consult with subject matter experts to interpret assessment results

The results of competency-based assessment of CTR skill may not be readily interpretable, particularly when no established criteria or rubric is associated with the assessment tool. In fact, many validated assessment tools do not prescribe how the resultant scores should be interpreted by respondents to better understand their own training needs or by training program administrators to enable programmatic improvements. In these cases it is important to consult with subject matter experts in clinical and translational research, psychometrics and statistical analysis while conducting analyses of the assessment results.

The need to consult with these types of subject matter experts is particularly acute with subjective and objective assessment tools. Subjective tests of knowledge and skill have been shown to be poorly correlated with objective measures ( Hodges, Regehr and Martin, 2001; Davis *et al*., 2006). There is evidence suggesting that while subjective measures of CTR knowledge and skill often increase between pre- and post-program tests the scores obtained through such objective tests do not increase at a similar rate ( Ellis *et al*., 2007; Cruser *et al*., 2010). Measurement and educational experts can help ensure that assessment results are interpreted in ways that are justified by the design and administration of the assessment instrument.

### Collect stakeholder feedback about options for programmatic improvement

An essential step of program evaluation involves sharing of evaluation results with stakeholder groups in order to facilitate collection of feedback about programmatic improvement ( Wandersman *et al*., 2000). For example, in the four overlapping and iterative phases of the Plan, Do, Check, and Act (PDCA) quality improvement cycle, the third stage typically involves studying the outcomes of a given initiative in ways that enable the articulation of what was learned through the implementation process ( Juran and DeFeo, 2010; Kleppinger and Ball, 2010). The involvement of stakeholders in this step of the process is critical to the rigorous evaluation of any CTR training program ( Trochim, Rubio and Thomas, 2013).

Reports of evaluation results should be customized to speak to stakeholder subgroups whenever it is not possible or productive to share the same report with all of them. For example, stakeholders with distinctive interests in the scientific content or pedagogical approach of a CTR training program may be most interested in reports showing how the results of competency-based assessment tools are being used to help participants identify and address their personal research learning challenges ( Chatterji, 2003). In contrast, stakeholders who value training programs as an institutional resource enabling the CTR enterprise may be more interested in the research careers or achievements of participants ( Frechtling and Sharp, 2002). Whenever possible thoroughly document stakeholder feedback so that it can be used to inform future discussions about programmatic improvement and impact.

## Conclusion

The guidelines presented here are intended to support the work of all clinical research professionals who are charged with the administration and evaluation of CTR training programs. In particular, this work fulfills a need for guidelines that clinical research investigators and administrators can follow to integrate competency assessment tools into their evaluation plans. Doing so will better enable research centers to collaborate with programmatic stakeholders efficiently and effectively in order to measure and improve the quality and impact of CTR training using the results of competency-based assessments of research knowledge and skill.

## Take Home Messages

• Effective training programs in clinical and translational research are critical to the development of the research workforce.
• Guidelines for integrating competency-based frameworks and assessments into program evaluations are needed to promote the quality and impact of research training programs.
• The stakeholders of clinical and translational research training programs should be routinely consulted throughout evaluation processes that involve competency frameworks and assessments.
• The systematic incorporation of competency-based approaches into evaluation plans facilitates the work of those developing, administering and evaluating research training programs.
• The use of validated competency assessments for programmatic evaluation is essential to the collection of reliable and relevant performance metrics.

## Notes On Contributors

All of the Co-authors contributed to the development of the guidelines presented in this work, informed the conclusions it advances and participated in all rounds of revisions required for submission.

Elias Samuels PhD, is the Manager of Evaluation at the Michigan Institute for Clinical and Health Research at the University of Michigan. ORCID ID: https://orcid.org/0000-0002-6725-3382

Phillip Anton Ianni PhD, is a Postdoctoral Research Fellow at the Michigan Institute for Clinical and Health Research at the University of Michigan. ORCID ID: https://orcid.org/0000-0003-1264-7322

Haejung Chung MA, is the Manager of Instructional Design and Technology at the Tufts Clinical and Translational Science Institute at Tufts University.

Brenda Eakin MS, is an Instructional Designer at the Michigan Institute for Clinical and Health Research at the University of Michigan. ORCID ID: https://orcid.org/0000-0002-7972-8621

Camille Martina PhD, is a Research Associate Professor in the departments of Public Health Sciences and of Emergency Medicine at the University of Rochester. ORCID ID: https://orcid.org/0000-0003-0523-7448

Susan Lynn Murphy ScD OTR/L, is an Associate Professor in the Department of Physical Medicine and Rehabilitation at the University of Michigan. ORCID ID: https://orcid.org/0000-0001-7924-0012

Carolynn Jones, DNP, MSPH, RN, FAAN is Associate Clinical Professor in the College of Medicine at The Ohio State University and Co-Director of Workforce Development for The Ohio State Center for Clinical Translational Science. ORCID ID: https://orcid.org/0000-0002-0669-7860
