# Supplementary material for: Guidelines for Evaluating Clinical Research Training using Competency Assessments
Source: MedEdPublish (2016). 2020 Sep 22;8:202. Originally published 2019 Nov 14. [Version 2] doi: 10.15694/mep.2019.000202.2 (PMC10712476; doi:10.15694/mep.2019.000202.2)
Supplement: Peer review history

# Reviewer report: Reviewer response for version 2

**DOI:** 10.21956/mep.20253.r31423

**Published:** 2020-10-02

**Authors:** David Bruce

**Affiliations:** 1 None

This review has been migrated. The reviewer awarded 4 stars out of 5 The authors are to be congratulated for tackling the complex issue of how to incorporate professional standards into clinical and translational research programmes. I think they have provided a logical set of steps for those charged with management and delivery of these programmes to consider and tailor to their own specific programmes.As a non specialist reader I though the paper provided sufficient background information about current CTR programmes to understand the need for the competency based framework and assessment programmes that they were proposing. I particularly liked the flow of their 12 guidelines which dealt with in turn; the faculty and stakeholders involved and their roles, the development of models, the creation of an assessment programme and the support for faculty to deliver this, the use of experts in helping interpretation and finally seeking stakeholder feedback. I wonder if this paper could be produced as a short pamphlet / guideline for those teaching and manging other CTR programmes. I would have liked to hear more about which assessments were considered and whither they were assessments of learning or assessment for learning. However – I appreciate this would lengthen the paper and may need to be described in a future paper. This paper will be of great interest to all involved in the research community.

Reviewer Expertise:

NA

No decision status is available

# Reviewer report: Reviewer response for version 2

**DOI:** 10.21956/mep.20253.r31422

**Published:** 2020-09-23

**Authors:** Trevor Gibbs

**Affiliations:** 1 AMEE

This review has been migrated. The reviewer awarded 5 stars out of 5 I congratulate the authors on their re-working of their paper.I found it easier to read this time and appreciate the improved clarity. I would recommend this paper to all those involved in curriculum planning and competency development

Reviewer Expertise:

NA

No decision status is available

# Reviewer report: Reviewer response for version 1

**DOI:** 10.21956/mep.18998.r27249

**Published:** 2020-03-16

**Authors:** Trevor Gibbs

**Affiliations:** 1 AMEE

This review has been migrated. The reviewer awarded 3 stars out of 5 An interesting although rather difficult paper to read. I personally found it difficult due to the interchange of the words evaluations and assessments, which to me have very distinct meanings.I would agree with all of my co-reviewer's comments and would add that I feel the main message(s) from this paper, i.e. the list of tips is lost in the complexity and lack of flow in the paper.This paper does cover an important area and one that has not been satisfactorily addressed, so I do congratulate the authors in attempting to apply logic to the question of how competency-based assessments used in Clinical and Translational Research (CTR) training can help inform the overall evaluation of a CTR programme. I would wonder how much importance one should place on the product outcomes as an evaluation measure of the programme.I do believe that this is a paper to recommend to those involved in evaluating research-training programmes, despite the reservations.

Reviewer Expertise:

NA

No decision status is available

# Reviewer report: Reviewer response for version 1

**DOI:** 10.21956/mep.18998.r27248

**Published:** 2019-11-30

**Authors:** David Bruce

**Affiliations:** 1 None

This review has been migrated. The reviewer awarded 3 stars out of 5 I thought that this was a complex and interesting paper where the authors consider how competency-based assessments used in Clinical and Translational Research (CTR) training can help inform the overall evaluation of a CTR programme. Guidelines for the evaluation of CTR programmes have been developed and propose that the professional development and training within the programmes for all staff and their level of knowledge and skills should be considered when such programmes are evaluated. The authors propose 12 guidelines to help administrators and training programme directors used competency-based assessment in their evaluations. The background to CRT programmes in the United States and funding across 50 research institutions (intuitions!) is outlined and the need for guidelines to help administrators and programme directors incorporate and make sense of competency based tests is proposed. At this stage in the paper I felt as a reader that some definitions and more clarity about CTR training programmes and who the CRT trainees are likely to be would have been helpful. As an example of what I mean about definitions, medical education programmes in the UK (undergraduate and postgraduate) are subject to quality control (by the providers) and quality management and quality assurance by regulators. This ensures that the standards for training are being met. I was unclear how this differed from the evaluations that this paper discusses. In respect of the training programmes within the CTR centres – I would like to have known the staff members in the training programmes and indeed if different training programmes were in place for different staff members. I assume some will be clinicians and some will be scientists. Are all in the one training programme / are the programmes clearly defined (learning outcomes defined and teaching and assessment programmes in place)? All this may be obvious to those involved in CTR programmes – but not clear to non-specialist readers. The paper then discusses each of the proposed guidelines. I counted 11 guidelines and not 12 as stated. Some of the guidelines proposed were general in nature - mapping of roles, data collection and use of IT platform - which matched previous guidelines for overall programme evaluation. A number of specific guidelines looked at competency based assessments. I felt that this part of the paper was less clear and more explanation was needed. The task for the administrators and training programme directors appeared to be considering what outcomes the programmes should deliver and the selection of validated competency assessment instruments. It now appeared to me as a reader that perhaps ad hoc training might be happening within the programmes and the evaluators were fitting competency assessments into this process. This may not be the case – but more explanation of the actual training programmes would be required to help explain these guidelines. I was also aware that the other factors normally considered when looking at the quality of an educational programme – such as the learning environment and how learners and their trainers are supported, did not seem to feature in these guidelines. This paper will be of interest to those working in CTR centres and those involved in CTR education and training. I think that for the general reader the authors need to provide more background and explanation of their proposals.

Reviewer Expertise:

NA

No decision status is available
